# Efficacy of metformin as an adjunct in periodontitis: A systematic analysis

**DOI:** 10.1016/j.jtumed.2026.03.007

**Published:** 2026-03-25

**Authors:** Muhammad K.H. Uddin, Aanya Naseem, Syeda M. Aziz, Saba Rafiq, Mehak A. Sattar, Muhammad S. Zafar, Bilquees Saba, Humaira Hassan

**Affiliations:** a Science of Dental Materials Department, Dr. Ishrat Ul Ebad Khan Institute of Oral Health Sciences, Dow University of Health Sciences, Pakistan; b Science of Dental Materials Department, School of Dental Care Professional, Dow University of Health Sciences Karachi, Sindh, Pakistan; c Science of Dental Materials Department, Dr. Ishrat Ul Ebad Khan Institute of Oral Health Sciences, Dow University of Health Sciences, Pakistan; d Department of Clinical Sciences, College of Dentistry, Ajman University, Ajman, United Arab Emirates; e Science of Dental Materials Department, Centre of Medical and Bio-allied Health Sciences Research, Ajman University, Ajman, United Arab Emirates; f Science of Dental Materials Department, School of Dentistry, Jordan University, Amman, Jordan; g Medicine and Allied, Department of Nephrology, Ziauddin University, Pakistan; h Science of Dental Materials Department, Panjwani Center for Molecular Medicine and Drug Research, ICCBS, University of Karachi, Pakistan

**Keywords:** Adjunctive therapy, Bone regeneration, Metformin, Periodontitis, RCTs

## Abstract

**Objective:**

To improve clinical and radiographic results in patients with periodontitis, this systematic review assessed the efficacy of metformin (MF) as an adjuvant to surgical and nonsurgical periodontal therapy.

**Methods:**

Patients with periodontitis (P), MF as an adjuvant (I), periodontal therapy alone, or placebo (C), and outcomes such as bone regeneration (O), clinical attachment level (CAL), and probing depth (PD), were evaluated with the PICO framework. Between June 15 and July 1, 2025, we searched for in vitro, in vivo, and randomized controlled trials (RCTs) published between June 2015 and June 2025 in PubMed, Google Scholar, ScienceDirect, and Semantic Scholar. Data were narratively synthesized according to MF concentration (0.5–1.5%), outcome measures, and patient type. The Cochrane tools SYRCLE and QUIN were used to assess risk of bias for RCTs, animal studies, and in vitro studies.

**Results:**

The analysis included 15 RCTs, 10 in vitro studies, and 8 in vivo studies among 4005 identified records. The most commonly used formulation was local MF gel. Follow-up times ranged from 1 to 9 months. MF, in contrast to scaling and root planing or placebo, produced vertical bone fill as high as 26.8%, a CAL gain of 1.5–2.7 mm, and a PD decrease of 1.5–3.4 mm. Experimental investigations had moderate risk of bias, whereas RCTs had low risk of bias.

**Conclusion:**

MF's osteogenic and anti-inflammatory qualities make it a promising adjuvant in periodontal therapy. However, the need for additional long-term research was highlighted by the study heterogeneity, limited follow-up periods, and a lack of meta-analysis.

## Introduction

Periodontitis, a long-term inflammatory condition affecting the periodontium, initially develops as gingivitis in response to microbial infection of the teeth, and leads to deterioration of tooth-supporting tissues and ultimately the loss of teeth.^1^^,^^2^ This condition affects 40–90% of the population globally and nearly 46% of the adult population in the United States.^3^ Pro-inflammatory cytokines strongly influence periodontal diseases. The elevated levels of nitric oxide, TNF-α, and PGE2 in the saliva in periodontitis might have potential value in detecting and treating periodontal disease. TNF-α stimulates the transcriptional factors NF-κB, c-Fos, and NFATc1, and plays a major role in osteoclast activation.^4^ Elevated PGE2 contributes to the breakdown of bone and supporting tissues.^5^ Conventional therapies, such as sub-gingival instrumentation and periodontal surgery, have been found to regenerate periodontal tissue. Despite current advances in therapeutic approaches, achieving adequate regeneration of periodontal tissue remains challenging.^6^

Metformin (MF), a second-generation biguanide, is a first-line oral medication for type 2 diabetes management, according to the American Diabetes Association.^6^ MF's therapeutic benefits include inhibiting hepatic gluconeogenesis through the AMPK pathway, and improving insulin sensitivity in muscle and adipose tissues.^7^ Beyond its effects in glycemic management, MF has anti-inflammatory properties.^8^ For example, it decreases oxidative stress and promotes the osteogenicity of human periodontal ligament cells by decreasing reactive oxygen species.^9^ Moreover, by activating the AMPK pathway, which elevates osteogenic markers such as Runx2 and promotes bone matrix formation, MF enhances osteogenic differentiation of mesenchymal stem cells, increases osteocalcin and osteogenic gene expression, and simultaneously decreases osteoclasts.^10^^,^^11^ Additionally, MF promotes phosphorylation of GSK3β, which in turn activates β-catenin and increases osteogenic gene expression, thus stimulating the Wnt/β-catenin pathway. Furthermore, MF might affect the Shh/Gli1 pathway, which promotes osteogenesis in coordination with AMPK and Wnt signaling.^11^ Therefore, MF is a promising option for periodontal regeneration.^12^

Although supplementary MF use in periodontal treatment has been examined in numerous studies, the results have been inconsistent because of variations in study design, patient demographics, administration methods (gel, film, or scaffold), and study outcomes (clinical, radiographic, or biochemical). In addition, current high-quality, multicenter trials with consistent methods and long-term follow-up are insufficient to warrant changes in periodontal treatment guidelines.

This review was aimed at identifying randomized controlled trials (RCTs) examining the radiographic and clinical efficacy of MF, compared with therapy alone or placebo, as a supplement to surgical or nonsurgical periodontal treatment, according to improvements in radiographic outcomes, clinical attachment level (CAL), and pocket depth (PD).

## Materials and Methods

### Review plan

This review complied with Preferred Reporting Items for Systematic Reviews and Meta-analysis (PRISMA) and Synthesis Without Meta-analysis (SWIM) reporting guidelines; however, it was not registered in a protocol database (e.g., PROSPERO),^13^^,^^14^ because of time constraints.

### Focused question

The following Population, Intervention, Comparison, Outcome (PICO) question was formulated^15^: in patients with periodontitis, what is the efficacy of MF as a supplement to surgical or nonsurgical periodontal treatment, compared with the same treatment alone or placebo, in improving PD reduction?

### Inclusion criteria (systematic review)

1) Study type: RCTs.
2) Study population: Systemically healthy adults (≥18 years of age) diagnosed with periodontitis.
3) Intervention types: MF (gel, biodegradable chip, or triple-layer mucoadhesive) as a supplement to surgical or nonsurgical periodontal treatment.
4) Comparison: The same periodontal treatment alone or with a placebo.
5) Outcomes: Primary: PD reduction, the distance between the gingival margin and the base of the pocket. Secondary: 1) CAL gain, the position of the soft tissue in relation to the cemento-enamel junction; 2) radiographic outcomes [reduced intra-bony defect (IBD) depth: a specific type of vertical bone loss in which the base of the pocket is below the crest of the surrounding bone, marginal bone loss (MBL): destruction of alveolar crestal bone around teeth or dental implants, and defective depth (DD): total extent of an IBD].

### Exclusion and inclusion criteria (systematic review)

Exclusion criteria:

1) Case reports, letters, or theses
2) Studies in participants with systemic diseases or medication use
3) Studies in participants with no MF use
4) Studies on MF use in non-periodontal models
5) Studies comparing MF and another adjunctive drug
6) Non-English articles

Inclusion criteria (supplementary evidence of biological plausibility):

1) In vitro and in vivo analyses of MF use in a periodontal framework.

### Search strategy

PubMed, Google Scholar, ScienceDirect, and Semantic Scholar were searched for studies published between January 2005 and June 2025. Articles in Web of Science, Embase, Cochrane Library, and grey literature were excluded, to avoid duplication and ensure methodological transparency.

Searches (conducted between June 15 and July 1, 2025) targeted all RCTs and in vivo/in vitro studies on MF's effects on periodontal tissues. Although the systematic review's primary focus was on RCTs, in vivo/in vitro studies were also screened systematically. However, the in vitro/vivo studies were not incorporated in the PRISMA flow or quantitative synthesis, but instead provided the basis for a narrative review to provide additional evidence. Searches were restricted to title, abstract, keywords, articles in English, and articles published in 2005–2025.

PubMed searches used MeSH and free text terms with Boolean operators (AND/OR):

1. (“Metformin” [MeSH] OR metformin OR “1,1-dimethylbiguanide hydrochloride” OR “dimethylbiguanide” OR Glucophage OR Glumetza OR Fortamet)

AND

(“Periodontitis” [MeSH] OR periodontitis OR “Gum disease” OR gingivitis OR pyorrhea)

Keywords for Google Scholar, ScienceDirect, and Semantic Scholar included the following:

1. “Metformin,” “Biguanides,” “Glucophage,” “Periodontitis,” “Periodontal diseases,” “Gum disease,” “pyorrhea,” and “RCT”

### Selection criteria

**Figure 1:**
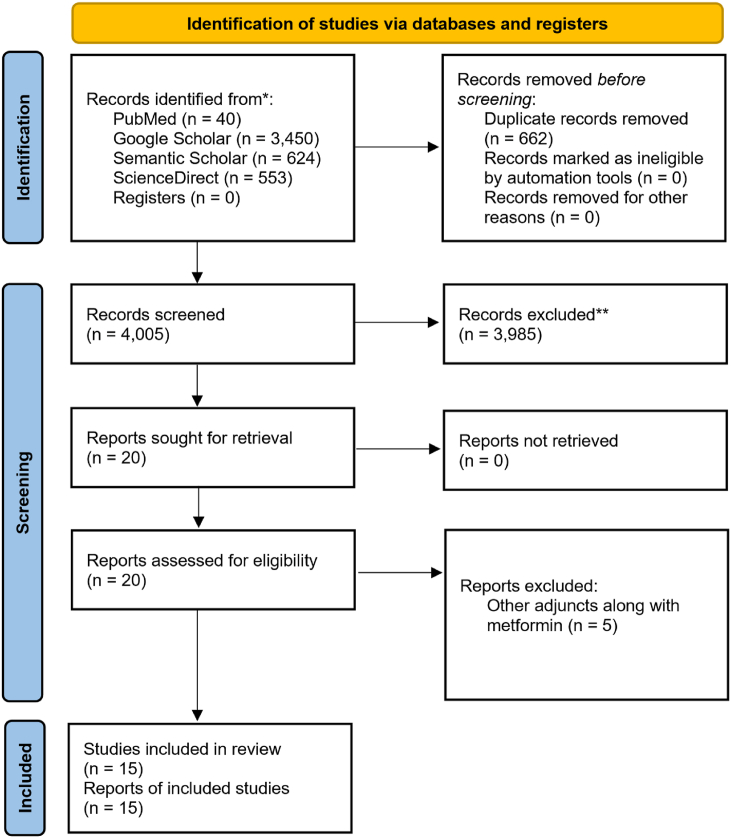
Prisma flowchart. This figure depicts the identification, screening, eligibility, and inclusion process for randomized controlled trials (RCTs) assessing the efficacy of the supplementary use of metformin with surgical or non-surgical periodontal treatment. The number of records obtained from electronic databases, duplicates eliminated, records screened, and studies excluded (including justifications) are summarized. Only RCTs obtained through this systematic flow were included in the PRISMA flow diagram. The in vivo and in vitro studies identified during the same electronic search were excluded from the PRISMA flow but were reviewed narratively to support the biological plausibility of MF's periodontal effects.

Four authors (S.R., A.N., M.A.S., and S.M.A.) performed calibration on a random subset, then independently screened titles and abstracts without blinding. An inter-reviewer reliability assessment indicated Cohen's kappa values of 1 for titles/abstracts and 0.72 for full texts after resolution of minor disagreements. Study eligibility was based on PICO and predefined criteria. Studies meeting these criteria were analyzed, but only RCTs were included in the PRISMA flowchart (Figure 1). The excluded studies are described in Table 1. In vitro, in vivo, and other studies were narratively reviewed to provide supportive evidence.

**Table 1:** Excluded studies.

Study	Reason for exclusion
1. **Bashir et al., 2022**	Use of another adjunctalong with metformin.
2. **Arslaan et al., 2022**	Use of another adjunctalong with metformin.
3. **Mirza et al., 2021**	Use of another adjunctalong with metformin.
4. **Kamel et al., 2021**	Use of another adjunctalong with metformin.
5. **Shah et al., 2022**	Use of another adjunctalong with metformin.

### Outcomes evaluated in pre-clinical studies

In vitro outcomes comprised effects on NLRP3 pyroptosis markers, the AMPK/NF-κB pathway, inflammatory markers (MMP-1, MMP-2, MMP-8, and IL-8), oxidative stress, senescence, the NPR3/MAPK pathway, osteogenesis, gene expression, the secretome, osteoclasts, the tAkt/Nrf2 pathway, and cementogenesis.

In vivo outcomes comprised effects on inflammatory cells, collagen degradation, osteoclast formation, alveolar bone loss, and the AMPK/SIRT1/autophagy pathway.

### Data collection

Four authors (A.N., S.M.A., S.R., and M.A.S.) independently extracted RCT data by using a custom Excel spreadsheet (title, authors, publication date, study design, population, MF type, comparator, outcomes, and follow-up) and resolved differences through discussion. In vitro and in vivo studies were narratively reviewed to provide supportive evidence. No assumptions were made regarding missing or unclear data, and neither author contact nor automation tools were used.

### Risk of bias evaluation

**Figure 2:**
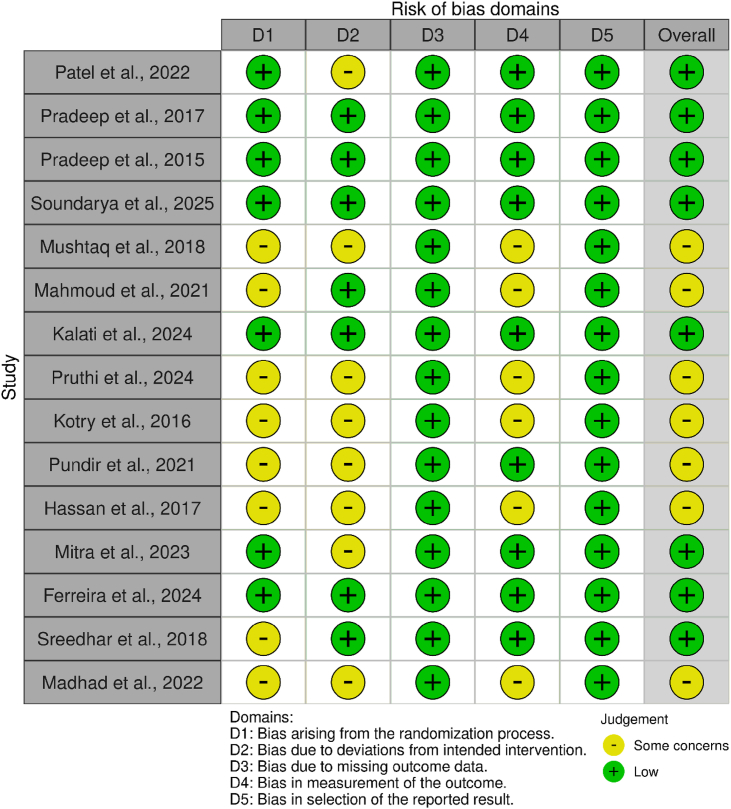
Traffic light plot for RCTs. Traffic light plot of RCTs. Most studies showed low risk across key domains, particularly domains 3 and 5. However, several studies, such as Pruthi et al., 2024; Kotry et al., 2016; and Madhad et al., 2022, were rated as having some concerns in domains 1 and 2.^7^^,^^27^^,^^28^ In contrast, Ferreira et al., 2024; Pradeep et al., 2017; Pradeep et al., 2015; Soundarya et al., 2025; and Kalati et al., 2024, were rated as having low risk across all five domains.^21^, ^22^, ^23^^,^^26^^,^^31^

**Figure 3:**
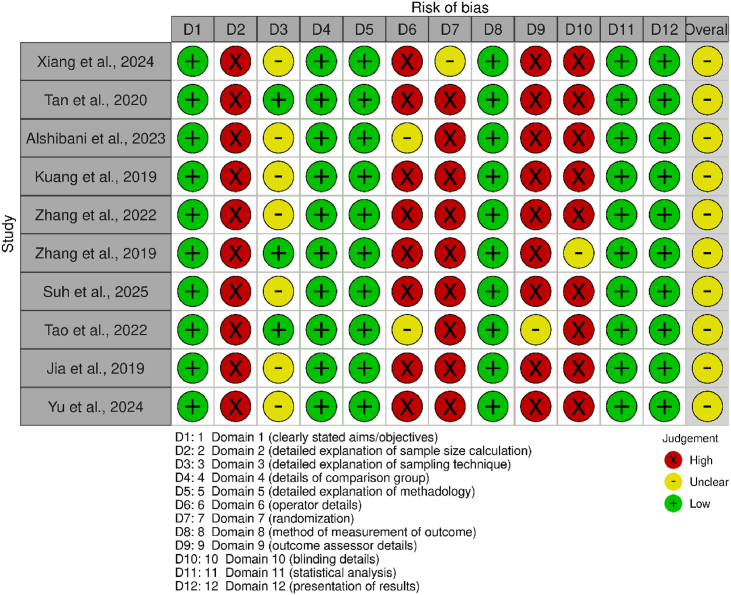
Traffic light plot for *in vitro* studies The QUIN tool, which contains 12 domains, was used to evaluate the methodological quality of ten studies. These domains included clearly stated aims/objectives (D1), sample size calculation (D2), sampling technique (D3), comparison group (D4), methodological description (D5), operator details (D6), randomization (D7), outcome measurement methods (D8), outcome assessor details (D9), blinding (D10), statistical analysis (D11), and result presentation (D12). Low risk (green), unclear risk (yellow), or high risk (red) were assigned to each domain. Clear study objectives, methodological explanations, outcome measurement strategies, comparison group, statistical analysis, and presentation of data were among the studies' shared strengths. However, flaws in blinding, randomization, sample size calculation, and details of the operator and outcome assessor were frequently found, thus suggesting the possibility of bias in several studies.

**Figure 4:**
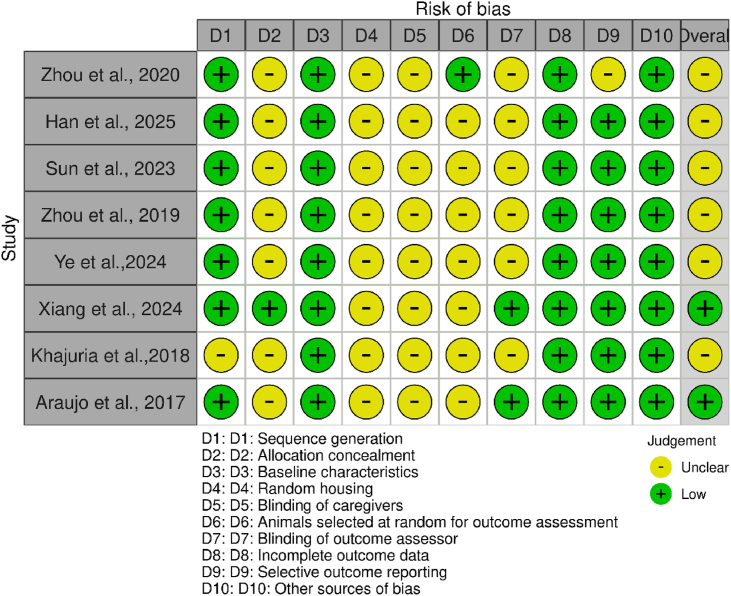
Traffic light plot for *in vivo* studies Color-coded plot for assessment of bias for in vivo studies with the SYRCLE tool. Ten risk-of-bias domains (D1–D10) were evaluated for each included study. Green circles indicate low risk of bias, whereas yellow circles indicate unclear risk. Most studies demonstrated low risk in baseline characteristics, completeness of outcome data, and outcome reporting, whereas the allocation concealment, randomization, and blinding domains were often unclear. Overall, the included studies exhibited moderate methodological reliability.

RCTs were assessed with the Cochrane criteria^16^ by two authors (A.N. and M.A.S.). The risk was rated as low, high, or unclear (Figure 2). Bias was evaluated at the study level, and study inclusion was unaffected, because no meta-analysis was conducted. In vitro studies were assessed with QUIN (S.M.A. and A.N.),^17^ and in vivo studies were assessed with SYRCLE (S.R. and M.A.S.)^18^ (plots in Figure 3, Figure 4). The pre-clinical assessments were reviewed separately as supportive evidence but were excluded from the central systematic review.

### Summary effect measures

RCT outcomes (PD reduction, CAL gain, IBD depth, and MBL decrease) were extracted as mean ± SD for the test and control groups. The mean differences and p-values are reported, when available. Because of heterogeneity in study design and follow-up, the effects were narratively synthesized rather than meta-analyzed.

### Standardized metrics and data transformation

All RCT outcomes (PD reduction, CAL gain, IBD depth, and MBL decrease) were continuous variables reported as mean ± SD for the test and control groups. Mean difference served as the standardized effect metric across studies. Because all studies used the same units (mm), no data transformation or unit conversion was required.

### Synthesis of results

Effect measures from each RCT were extracted for PD reduction, CAL gain, and decrease in IBD depth and MBL, reported as mean ± SD for the test and control groups. Mean differences and p-values are reported, when available. On the basis of the comparative mean values and statistical significance, the direction and magnitude of effects were narratively synthesized.

### Grouping of studies for synthesis

Studies were grouped narratively by clinical characteristics. RCTs were grouped into (1) surgical treatment with supplementary MF and (2) non-surgical treatment with supplementary MF. Further subgrouping was performed according to localized periodontal defects. This framework enabled comparison of MF's effects across treatment characteristics. In vivo/in vitro studies were reviewed separately to support the biological plausibility of the observed clinical effects.

### Criteria for prioritizing results

RCTs were prioritized, because they best addressed the PICO question. In vitro and in vivo studies were used only to provide mechanistic support, and were narratively summarized but not included in the PRISMA flow or core synthesis. Among the RCTs, greater importance was placed on studies with a lower risk of bias and sufficient follow-up.

### Investigation of heterogeneity in reported effects

Heterogeneity was assessed narratively, because meta-analysis was not feasible. Differences in treatment type (surgical and nonsurgical), MF dose, delivery, and the direction/magnitude of effects were compared to identify variability. Methodological factors such as sample size and risk of bias were also considered in interpreting discrepancies.

### Reporting bias assessment

Because meta-analysis was not conducted, formal reporting bias tests were not performed. Comprehensive research helped reduce publication bias, although the possibility of selective outcome reporting cannot be fully excluded.

### Certainty assessment

Certainty of evidence regarding the reduction in PD, and gain in CAL and radiographic outcomes, reported in each RCT was assessed with Grading of Recommendations, Assessment, Development and Evaluation (GRADE) criteria with Gradepro GDT,^19^ considering risk of bias, inconsistency, indirectness, imprecision, and publication bias. Outcomes were rated as high, moderate, low, or very low. Two authors (A.N. and M.A.S.) assessed certainty independently, and disagreements were resolved through discussion.

### Data presentation

Data are presented in tables and figures. RCT tables include study design, intervention, control, follow-up, mean ± SD, mean differences, p-values, and effect direction. In vivo/in vitro tables include design, intervention, delivery, cell types, and outcomes. GRADE and risk-of-bias assessments are presented in tables and traffic light figures. The table of excluded studies and PRISMA flow diagram documented the selection process.

### Additional analyses

No sensitivity, subgroup, or additional analyses (e.g., meta-regression) were performed.

## Results

### Search results

A total of 4005 articles were screened after duplicate removal, 3985 of which were excluded on the basis of their title/abstract. A comprehensive analysis of 20 articles was conducted. After exclusion of five more articles for the reasons stated in Table 2, 15 studies^5^^,^^7^^,^^20^, ^21^, ^22^, ^23^, ^24^, ^25^, ^26^, ^27^, ^28^, ^29^, ^30^, ^31^, ^32^ were selected for this review.

**Table 2:** Summary of RCTs on metformin in periodontal therapy.

S. No	Study ID	Design (Parallel/Split Mouth)	n (Patients)	Age (Range)	Comorbidities	Disease Definition	Therapy Details	MF Concentration	Mode of Delivery	Duration	Outcomes (PD, CAL, or Bone Fill)
1,	**Mahmoud G. et al., 2021**	Parallel group	10/10 (total 20 patients)	25–30 years	Systemically healthy	Severe chronic periodontitis with intra-bony defects (PD ≥ 5 mm after phase I therapy)	Surgical (OFD). Phase I SRP under LA. Surgery: Flap elevation, debridement, SRP, rinsing. Single application.	1% MF gel	Locally delivered gel (applied to the defect)	9 months	PD, CAL, marginal bone loss (MBL)
2.	**Pradeep et al. 2015**	Parallel group	30/30 (total 60 completed)	25–50 years	Systemically healthy	Chronic periodontitis; PD ≥ 5 mm, CAL ≥4 mm, and vertical bone loss ≥3 mm	Non-surgical SRP (full mouth in two sessions) under LA. Post-operative: Avoid hard foods and interdental aids for 1 week. No antibiotics. Single application.	1% MF gel	Locally delivered gel	6 months	PD, CAL, intra-bony defect depth (IBD
3.	**Pradeep et al. 2017**	Parallel group	32/∼32 (total 64 completed)	30–50 years	Systemically healthy	Chronic generalized periodontitis; PD ≥ 5 mm, CAL ≥4 mm, and vertical bone loss ≥3 mm	Non-surgical SRP (full-mouth SRP) under LA (if necessary). Post-operative: Avoid hard foods and interdental aids for 1 week. No antibiotics. Repeated application (BL, 3 M, 6 M).	1% MF gel	Locally delivered gel	9 months	PD, CAL, IBD depth reduction (DDR%)
4.	**Patil et al. 2022**	Split mouth	15/15 sites (total 15 patients)	25–60 years	Systemically healthy	Chronic periodontitis with IBDs; IBD ≥3 mm, CAL ≥4 mm, and PPD ≥5 mm	Non-surgical SRP + curettage. Full-mouth SRP/curettage. Post-operative: no flossing for 10 days or chemotherapeutic mouth rinse. Single application.	1.5% MF gel	Locally delivered gel	6 months	PD, CAL, infra-bony defect depth (IBD fill)
5.	**Mushtaq et al. 2018**	Parallel group	15/15 (total 30 patients)	25–55 years	Systemically healthy	Chronic periodontitis: PD ≥ 5 mm and CAL ≥4 mm	Non-surgical SRP (complete phase I therapy). Post-operative: Avoid hard/sticky foods, brushing treated areas, or interdental aids for 1 week. No antibiotics. Single application.	1% MF gel	Locally delivered gel	3 months	PD, CAL
6.	**Soundarya et al. 2025**	Parallel group (split-mouth method)	26/24 sites (total 25 patients)	30–60 years	Systemically healthy	Stage II, grade B periodontitis; sites with PPD >3 mm and ≤5 mm and CAL 3–4 mm	Non-surgical SRP (full SRP performed). Post-operative: Avoid chewing on the treated side or touching the site. Single application.	1% MF (chip)	Locally delivered biodegradable chip	3 months	PD, CAL
7.	**Pruthi et al. May 2024**	Parallel group	10/10 (total 20 patients)	20–50 years	Systemically healthy	Stage I/II, grade A/B periodontitis; minimum 8 teeth with PPD of 5–7 mm	Non-surgical SRP (complete phase I). LDD followed by Coe Pak dressing (7 days). Post-operative: Avoid hard/sticky food and antibiotics. Single application.	1% MF gel	Locally delivered gel	3 months	PD, CAL
8.	**Annaji Sreedhar et al. 2022**	Split mouth	15/15 (total 15 patients)	34–64 years	Systemically healthy	Chronic periodontitis; at least one site with PPD ≥5 mm in three quadrants	Non-surgical SRP (full-mouth SRP). Post-operative: Avoid hard/sticky food for 1 week; normal rinsing/brushing. G2: Single dose; G3: Multiple doses (days 1 and 30).	1% MF gel	Locally delivered gel	3 months	PD, CAL
9.	**Madhad et al. 2022**	Parallel group	30/30 (total 60 patients)	25–55 years	Systemically healthy	Chronic generalized periodontitis; PD ≥ 5 mm and CAL ≥4 mm	Non-surgical SRP (full-mouth oral prophylaxis). Periodontal dressing applied. Post-operative: Avoid hard/sticky foods or brushing near treated areas for 1 week. Single application.	1% MF gel	Locally delivered gel	6 months	PD, CAL
10.	**Mitra D. K. et al. 2023**	Split mouth	10/10 sites (total 10 participants)	Not reported	Systemically healthy	Chronic periodontitis; at least two IBDs with PPD ≥5 mm after phase I SRP	Surgical (OFD) + DFDBA graft. LA (2% lignocaine). Post-operative: Sutures/dressing removed (7 days); antibiotics (amoxicillin/metronidazole/ibuprofen) prescribed; 0.2% CHX mouthwash (2 weeks). Single application.	1% MF gel	Local gel + graft (mixed with DFDBA)	9 months	PD, relative attachment level (RAL), radiographic defect depth (DD), crestal bone height (CBH)
11.	**Hasan et al. 2017**	Parallel group (N = 4)	10 (MF Gel)/10 (gRP)/10 (MW)/10 (control) (total 40 patients)	Above 20 years range NR	Systemically healthy	Gingivitis and periodontitis; diagnosis based on PPD and AL measured	Non-surgical SRP (per quadrant per week for 4 weeks). MF applied after 48 h of every SRP session (gel group).	1% MF gel	Locally delivered gel	1 month	PD, attachment level (AL)
12.	**Pundir et al. 2021**	Split mouth design; parallel group (sites)	40/40 sites (total 39 patients completed)	Above 25 years range NR	Systemically healthy	Chronic periodontitis; pocket depth ≥5 mm (PPD and radiographic examination)	Non-surgical SRP (initial SRP). Post-operative: Periodontal pack (7 days); avoid hard/sticky foods, brushing near treated areas, or interdental aids for 1 week. No medications. Single application.	1% MF gel	Locally delivered gel	9 months	PD, CAL
13.	**Kotry et al. 2016**	Parallel group	10/10 sites (total 20 patients)	36–55 years	Systemically healthy	Moderate–severe chronic periodontitis; PPD ≥5 mm and CAL ≤7 mm	Non-surgical SRP. Post-operative: Avoid hard/sticky foods, brushing treated areas, or interdental aids for 1 week. Single application.	1%MF (film)	Locally delivered gel triple-layer mucoadhesive film	6 months	PD CAL, radiographic IBD depth
14.	**Ferreira et al. 2024**	Parallel group	20/19 (total 39 patients)	18–70 years	**Type II diabetes mellitus** (all taking systemic MF)	Periodontitis (PD) stages 1, 2, 3, or 4, grade B or C; sites with PD ≥ 4 mm treated	Non-surgical SRP. Antibiotic prophylaxis (2 g amoxicillin) 1 h before appointment. Applied once.	1% MF gel	Locally delivered gel	6 months	PD, CLI
15.	**Arbabi Kalati et al. 2024**	Parallel group	18/18 (total 36 completed)	Range NR	Systemically healthy	Severe chronic periodontitis; PD ≥ 6 mm and CAL ≥5 mm	Non-surgical SRP (full-mouth SRP). Applied initially at baseline and a second time after 2 months (retreatment).	1% MF gel	Locally delivered gel	4 months	PD, CAL

Abbreviations: NR = Not Reported, SRP = Scaling and Root Planning, MF = Metformin, OFD = Open Flap Debridement, PD = Probing Depth, CAL = Clinical Attachment Level, AL = Attachment Level, RAL = Relative Attachment Level, CLI = Clinical Loss of Insertion, MBL = Marginal Bone Loss, IBD = Intra-bony Defect Depth, DD = Defect Depth, LDD = Local Drug Delivery.

### Summary of included studies

Fifteen RCTs (June 2015 to June 2025) were selected (key findings summarized in Table 2). These studies evaluated the adjunctive effects of MF in patients with periodontitis undergoing surgical or non-surgical periodontal intervention. The RCTs had heterogeneous sample sizes (20–90 participants) and follow-up intervals (1–9 months). The delivery approaches differed. Most studies examined local MF (gel) administration at concentrations of 0.5%, 1%, or 1.5%. Some studies explored advanced systems such as chips and films,^7^^,^^23^ whereas others integrated MF with bone.^30^ Most trials reported statistically significant improvements in clinical outcomes, including reductions in PD, CAL gain, and radiographic outcomes (IBD, MBL, and DD), for MF compared with SRP alone or placebo. Comprehensive statistical data are presented in Table 3A (non-surgical RCTs) and Table 3B (surgical RCTs), detailing baseline and post-treatment mean PD, CAL, and bone outcomes in the intervention and control groups, along with p-values and mean differences.

**Table 3A:** Summary of all outcomes related to non-surgical therapies

S. No	Study	Outcome	Baseline (MF) mm ± SD	Baseline (Control) ± SD	After Treatment (MF) ± SD	After Treatment (Control) ± SD	p-value (Significance), MF vs Control	Improvement (MF)	Improvement (Control)	Mean Difference (MF - Control)	95% Confidence Interval
1.	Pradeep et al., 2015	PD	8.03 ± 0.76	7.96 ± 1.09	4.06 ± 0.78	5.96 ± 0.92	P < 0.001	3.97	2.00	1.97	Not reported
		CAL	6.23 ± 0.77	6.13 ± 0.93	2.16 ± 0.746	4.7 ± 0.65	P < 0.001	4.07	1.43	2.64	
		IBD	5.01 ± 0.66	4.7 ± 0.50	3.65 ± 0.52	4.53 ± 0.41	P < 0.001	1.36	0.17	1.19	
2.	Pradeep et al., 2017	PD	6.6 ± 0.46.46	6.7 ± 0.46	3.6 ± 0.86	5.7 ± 0.27	P < 0.05 or P < 0.001	3.0	2.00	2.00	Not reported
		CAL	6.4 ± 0.91	6.0 ± 0.14	2.5 ± 0.87	6.0 ± 0.14	P < 0.05 or P < 0.001	3.9	0.6	3.30	
		IBD	5.0 ± 0.64	4.8 ± 0.49	3.5 ± 0.46	4.7 ± 0.50	P < 0.05 or P < 0.001	1.50	0.10	1.40	
3.	Patil et al., 2022	PD	5.33 ± 0.617	5.2 ± 0.414	2.773 ± 0.458	3.667 ± 0.488	P = 0.000	2.557	1.533	1.024	Not reported
		CAL	5.33 ± 0.617	5.2 ± 0.414	2.773 ± 0.458	3.667 ± 0.488	P = 0.000	2.557	1.533	1.024	
		IBD	3.667 ± 0.816	3.4 ± 0.632	0.533 ± 0.64	2.133 ± 0.516	P = 0.000	3.1345	1.267	1.867	
4.	Mushtaq et al., 2018	PD	6.44 ± 0.38	6.39 ± 0.48	2.13 ± 0.38	4.19 ± 0.51	P = 0.001	4.31	2.20	2.11	Not reported
		CAL	6.25 ± 0.23	6.21 ± 0.43	2.17 ± 0.29	4.40 ± 0.74	P = 0.001	4.08	1.81	2.27	
5.	Soundarya et al., 2025	PD	6.04 ± 0.79	6.16 ± 0.80	4.20 ± 0.91	4.56 ± 0.82	P = 0.2404 (Not significant)	1.84	1.60	0.24	Not reported
		CAL	6.00 ± 0.71	6.16 ± 0.80	3.76 ± 0.60	5.12 ± 0.78	P = 0.0001.	2.24	1.04	1.20	
6.	Pruthi et al., 2024	PD	4.80 ± 0.83	4.20 ± 0.83	3.40 ± 0.89	2.80 ± 0.83	P = 0.003	1.40	1.40	0.00	Not reported
		CAL	4.80 ± 0.83	4.40 ± 0.89	3.40 ± 0.89	3.80 ± 0.83	P = 0.005	1.40	0.60	0.80	
7.	Annaji Sreedhar et al., 2018	PD	5.80 ± 0.676	5.93 ± 0.593	4.26 ± 0.883	5.26 ± 0.703	P = 0.003	1.54	0.67	0.87	
		CAL	8.80 ± 0.676	8.86 ± 0.516	7.13 ± 0.990	8.20 ± 0.676	P = 0.002	1.67	0.66	1.01	
8.	Mahad et al., 2022	PD	3.0667	3.2500	1.3133	2.4600	P < 0.001	1.7534	0.7900	0.9634	Not reported
		CAL	7.8967	7.2967	4.7367	6.0000	P < 0.001	3.1600	1.2967	1.8633	
9.	Hasan et al., 2017	PD	4.28 ± 1.04	3.90 ± 0.83	2.57 ± 1.07	3.77 ± 0.95	p ≤ 0.001	1.71	0.13	1.58	Not reported
		AL	4.43 ± 1.00	3.79 ± 0.55	2.74 ± 1.01	3.57 ± 1.00	p ≤ 0.001	1.69	0.22	1.47	
10.	Pundir et al., 2021	PD	6.48 ± 0.87	6.78 ± 0.84	2.16 ± 0.31	4.08 ± 0.79	P < 0.05	4.32	2.70	1.62	Not reported
		CAL	6.20 ± 0.07	6.78 ± 0.7	2.74 ± 0.47	4.12 ± 0.61	P < 0.05	3.46	2.66	0.80	
11.	Kotry et al., 2016	PD	6.4 ± 1.07	6.2 ± 1.03	3.20 ± 0.92	4.40 ± 0.52	P = 0.0020	3.20	1.80	1.40	Not reported
		CAL	6.20 ± 0.07	5.40 ± 1.07	2.74 ± 0.47	3.50 ± 0.97	P = 0.0465	2.70	1.90	0.80	
		IBD	4.40 ± 0.97	4.80 ± 1.14	3.20 ± 0.92	4.40 ± 1.17	P = 0.0203	1.20	0.40	0.80	
12.	Ferreira et al., 2024	PD	2.3 ± 0.6	1.9 ± 0.4	1.8 ± 0.5	1.7 ± 0.4	p < 0.05	0.5	0.2	0.30	Not reported
13.	Arbabi Kalati et al., 2024	PD	7.33 ± 1.96	6.79 ± 1.53	3.83 ± 0.96	4.92 ± 1.32	P = 0.007	3.50	1.87	1.63	Not reported
		CAL	6.68 ± 0.81	6.35 ± 0.77	3.71 ± 1.14	4.63 ± 1.01	P = 0.014	2.97	1.72	1.25	

Note: Madhad et al., 2022, did not report any SD values. PD = Probing Depth, CAL = Clinical Attachment Level, IBD = Infra-bony Depth, MBL = Marginal Bone Loss, DDR = Defect Depth Reduction, MF = Metformin.

**Table 3B:** Summary of all outcomes related to surgical therapies.

S. No	Study	Outcome	Baseline (MF) mm ± SD	Baseline (Control) ± SD	After Treatment (MF) ± SD	After Treatment (Control) ± SD	p-value (Significance), MF vs Control	Improvement (MF)	Improvement (Control)	Mean Difference (MF - Control)	95%Confidence Interval
1,	Mahmoud G.et al., 2021	PD	5.64 ± 0.08	5.54 ± 0.14	3.41 ± 0.44	4.19 ± 0.14	p = 0.00	2.23	1.35	0.88	Not reported
		CAL	4.2 ± 0.10	4.31 ± 0.17	2.7 ± 0.15	3.30 ± 0.24	p = 0.00	1.50	1.01	0.49	
		MBL	3.56 ± 0.28	3.55 ± 0.06	2.0 ± 0.23	2.70 ± 0.11	p = 0.00	1.56	0.85	0.71	
2.	Mitra et al., 2023	PD	6.4	6.1	3.6	3.2	P = 0.749 (not significant)	2.8	2.9	0.40	N/A
		DDR	7.91	7.84	1.19	1.05	P = 0.856 (not significant)	6.72	6.79	0.14	

Note: Mitra et al., 2023, did not report any SD values. PD = Probing Depth, CAL = Clinical Attachment Level, IBD = Infra-bony Depth, MBL = Marginal Bone Loss, DDR = Defect Depth Reduction, MF = Metformin.

### Risk of bias assessment

Risk of bias across the 15 RCTs was assessed with the Cochrane Rob2.0 tool. Eight studies showed low risk of bias, whereas the other seven posed some concerns due primarily to poor blinding, inadequate description of the randomization process, and limitations in outcome measurement. Importantly, no studies were rated as having high risk. A full evaluation is shown in Figure 2.

### Clinical outcomes (RCTs)

#### Non-surgical therapy

Thirteen RCTs investigated MF as an adjunct to SRP in non-surgical periodontal therapy. These studies consistently demonstrated improved clinical and radiographic parameters when MF was used in conjunction with SRP, compared with SRP or placebo alone. All 13 reported PD^5^^,^^7^^,^^20^, ^21^, ^22^, ^23^, ^24^^,^^26^, ^27^, ^28^, ^29^^,^^31^^,^^32^ outcomes, whereas 12 reported CAL,^5^^,^^7^^,^^20^, ^21^, ^22^, ^23^, ^24^^,^^26^, ^27^, ^28^, ^29^^,^^32^ and only 4 documented radiographic outcomes.^7^^,^^20^, ^21^, ^22^

(i) Probing Depth

Most non-surgical trials indicated a statistically significant reduction in PD favoring MF adjuncts. The mean PD improvements in MF-treated sites ranged from 0.5 mm to 4.31 mm, whereas the control groups showed decreases of 0.2 mm–2.70 mm. Notably, Mushtaq et al., 2018,^24^ and Pundir et al., 2021,^29^ indicated PD improvements exceeding 4.0 mm with 1% MF gel. In patients with diabetes, Ferreira et al., 2024,^31^ reported similar benefits, thereby supporting MF's efficacy in conditions compromised by impaired healing.

(ii) Clinical Attachment Level

Adjunctive MF led to significantly greater CAL gain, ranging from 1.5 mm to 4.09 mm, than SRP alone. This improvement was sustained over follow-up periods of 3–9 months, and clinical responses were evident as early as 1 month.

(iii) Radiographic Bone Outcomes

Among 13 RCTs, only 4^7^^,^^20^, ^21^, ^22^ documented radiographic bone outcomes associated with IBD reduction or bone fill. All four demonstrated significant improvements in the MF-treated groups vs controls. For example, Patil et al., 2022,^20^ demonstrated this effect through a greater reduction in mean IBD in the MF-treated group than the control, thus indicating that locally delivered 1.5% MF gel improves radiographic outcomes.

#### Surgical therapy

Two RCTs explored the efficacy of MF as an adjunct to periodontal flap or regenerative surgical therapy.^25^^,^^30^

(i) Probing Depth and Clinical Attachment Level

Surgical intervention supplemented with MF demonstrated a reduction in PD ranging from 3.41 mm to 3.6 mm. Mahmoud et al., 2021, reported CAL values and observed a decrease from 4.2 ± 0.10 to 2.7 ± 0.15 in the MF group, and from 4.31 mm to 3.30 mm in the control group, thus indicating a superior CAL gain with adjunctive MF.

(ii) Radiographic Outcomes

Both reported radiographic outcomes. Mahmoud et al., 2021,^25^ assessed MBL and revealed superior improvement in the MF group than the control group after flap surgery and 1% MF gel application. These findings highlight MF's enhanced bone-preserving effects during regenerative flap procedures. Additionally, greater radiographic bone fill was observed in the MF + demineralized freeze-dried bone allograft (DFDBA) group than the group with DFDBA alone, thereby affirming MF's osteogenic and regenerative potential in surgical contexts. Overall, surgical adjunct studies indicated that MF either surpassed outcomes of surgery alone or matched those of standard regenerative approaches. In contrast, non-surgical approaches consistently produced quantitatively greater enhancements in clinical parameters: non-surgical studies exhibited larger PD reductions and CAL gains (e.g., 4.31 mm PD reduction and 4.07 mm CAL gain). This finding differed from the surgical outcomes (e.g., 2.23 mm PD reduction and 1.5 mm CAL gain).

#### Localized defects

All RCTs consistently focused on localized periodontal defects, even when participants were diagnosed with generalized chronic periodontitis. In each study, MF was administered locally, to specific defect sites that met predefined criteria for advanced localized disease. For example, Pradeep et al., 2017,^21^ and Madhad et al., 2022,^28^ treated individualized defect sites per patient despite a generalized diagnosis.

### Assessment of finding and certainty of evidence

The GRADE framework (Table 4) was used to assess the certainty of evidence. Supplementary MF treatment, compared with controls, improved the reductions in PD and gains in CAL. Radiographic data suggested possible bone regeneration benefits; however, the certainty of evidence remained moderate for PD and CAL and low for radiographic measures.

**Table 4:** Summary of certainty of evidence (GRADE).

Summary of findings (GRADE) for metformin as an adjunct to periodontal therapy vs placebo or standard periodontal therapy alone in patients with periodontitis						
Outcome andFollow-up	Patients(Studies), N	RelativeEffect(95% CI)	Absolute Effects (95% CI)			Certainty
			Placebo or StandardPeriodontal TherapyAlone	MetforminAdjunct toPeriodontalTherapy	Difference	
Probing depth	542 (15 RCTs)	–	Not pooled	Not pooled	Not pooled	⨁⨁⨁◯ Moderate^a^^,^^b^
Clinical attachment loss	486 (13 RCTs)	–	Not pooled	Not pooled	Not pooled	⨁⨁⨁◯ Moderate^c^
Radiographic outcomes	220 (6 RCTs)	–	Not pooled	Not pooled	Not pooled	⨁⨁◯◯ Low^d^^,^^e^

**CI:** confidence interval.

a Study on patients with diabetes (Ferreira et al., 2024).

b Missing standard deviation (SD) in two studies (Mahdad et al., 2022, and Mitra et al., 2023).

c Missing SD in Mahdad et al., 2022.

d Variation in delivery systems and surgical vs non-surgical context.

e Small sample size; missing SD in one study (Mitra et al., 2023).

### In vitro studies

Ten studies investigated the mechanisms through which MF supports periodontal regeneration and modulates inflammation. The findings are organized by cell type for clarity and are summarized in Table 5. The risk of bias for in vitro studies was assessed with the QUIN tool (Figure 3).

**Table 5:** Summary of *in vitro* studies on metformin in periodontal therapy.

Study	Cell Type Used	MF Dose/Concentration	Outcomes Measured	Key Findings
**Xiang et al., 2024**	hPDLSCs	10 μM,50 μM,100 μM	CCK-8 and migration, osteogenic differentiation, *IL-6*, and *IL-8*	MF treatment significantly increased the yield of EVs and generated metformin-treated EVs with superior effects on PDLSC proliferation, migration, osteogenic differentiation, and anti-inflammatory potential in vitro.
**Alshibani et al., 2023**	HGFs	500 μM,1000 μM, 2000 μM	MMP-1, MMP-2, MMP-8, and IL-8 expression	MF reduced inflammatory cytokines and protected soft tissue.
**Kuang et al., 2019**	PDLSCs	100 μM	Oxidative stress markers, senescence	MF prevented oxidative-stress-induced senescence in PDLSCs.
**Zhang et al., 2022**	PDLSCs (diabetic model)	10–1000 μM	Osteogenesis and NPR3/MAPK signaling	MF reversed high-glucose suppression of osteogenesis by inhibiting the MAPK pathway.
**Zhang et al., 2019**	PDLSCs	10 μM, 50 μM, 100 μM	Proliferation, migration, and osteogenic differentiation	MF enhanced key regenerative functions of PDLSCs.
**Suh et al., 2025**	PDLSCs (via Met-CM Secretome)	50 μM	Transcriptome, secretome, and regenerative signaling	MF-CM reversed inflammation-induced suppression and promoted regeneration.
**Tao et al., 2022**	Periodontal ligament fibroblasts	8–1000 μM	Osteoclast formation, mineralization, and inflammatory mediators	MF inhibited osteoclastogenesis and promoted osteogenesis in PDLFs.
**Jia et al., 2020**	PDLSCs	100 μM, 500 μM, 1000 μM	Akt/Nrf2 pathway, oxidative stress, and osteogenic differentiation	MF promoted osteogenic differentiation and decreased oxidative stress.
**Yu et al., 2025**	hPDLSCs (dental resin system)	7.5% (w/w)	Osteogenic and cementogenic differentiation	MF-resin composite enhanced differentiation, thus supporting regenerative applications.
**Tan et al., 2020**	hPDLCs	50,000 μM	**IL-1β, IL-18, NLRP3, Caspase-1, and CCK-8 assays**	MF had anti-inflammatory effects via **downregulation of NLRP3 inflammasomes and NF-κB/TNF-α pathways**.

Abbreviations: hPDLCs = Human Periodontal Ligament Stem Cells, HGFs = Human Gingival Fibroblasts, PDLFs = Periodontal Ligament Fibroblasts, HPDLC = Human Periodontal Ligament Cells, EVs = Extracellular Vesicles, CCK-8:Cell Counting Kit-8, NLRP3 = NOD-like Receptor Family Pyrin Domain-containing 3, MAPK = Mitogen-activated Protein Kinase, MF = Metformin.

#### Periodontal ligament stem cells (PDLSCs)

Studies on PDLSCs uniformly demonstrated MF's potent regenerative and protective benefits. Zhang et al., 2019,^33^ observed that MF increased proliferation, migration, and osteogenic differentiation, whereas Jia et al., 2020,^34^ identified Akt/Nrf2 pathway activation as a safeguard against oxidative stress. In high-glucose settings, Zhang et al., 2022,^35^ demonstrated that MF restores osteogenesis through NPR3/MAPK suppression. Additionally, Kuang et al., 2019,^36^ showed that MF inhibits oxidative stress-induced senescence, thereby sustaining PDLSC functionality.

#### Human Periodontal Ligament Stem Cells (hPDLSCs)

Yu et al., 2025,^37^ developed an MF-infused dental resin promoting osteogenic and cementogenic differentiation in hPDLSCs, thus enabling localized MF delivery. Suh et al., 2025,^38^ demonstrated that conditioned medium from MF-pretreated PDLSCs amplifies regenerative signaling while countering inflammation-mediated inhibition. Xiang et al., 2024,^6^ showed that MF enhances intrinsic osteogenesis in hPDLSCs and increases the production and bioactivity of PDLSC-derived extracellular vesicles, which exert superior regenerative and anti-inflammatory effects, thereby reflecting both direct and EV-mediated mechanisms.

#### Human periodontal ligament cells (hPDLCs)

Tan et al., 2020,^1^ demonstrated that MF substantially mitigated inflammation induced by *Porphyromonas gingivalis* lipopolysaccharide in hPDLCs. This protective effect arose from MF's suppression of NLRP3 inflammasome activation and decreased the release of IL-1β and IL-18. Transcriptomic analysis additionally revealed MF's regulation of NF-κB and TNF-α pathways, thus underscoring a multifaceted strategy for modulating the inflammatory response in periodontitis.

#### Periodontal ligament fibroblasts (PDLFs)

In a study by Tao et al., 2020,^39^ MF modulated the signaling activity of PDLFs in a selective manner without changing osteogenic function. MF was found to inhibit PDLF-mediated osteoclastogenesis by downregulating genes associated with osteoclastogenesis, including RANKL and M-CSF.

#### Human Gingival Fibroblasts (hGFs)

Alshibani et al., 2023,^40^ examined the effects of MF on inflamed hGFs. MF was found to decrease the expression of matrix metalloproteinases and interleukin-8 **(**IL-8), and therefore might help stabilize soft tissues and calm the immune response, thus facilitating periodontal wound healing. Overall, in vitro data suggest that MF has a range of regenerative and anti-inflammatory actions.

### In vivo studies

#### Effects of MF on oxidative stress and inflammation

Several preclinical studies have shown that MF is highly effective in treating periodontitis. Table 6 summarizes the results of these in vivo investigations. In animal models of periodontal disease, MF has been demonstrated to lessen bone loss, oxidative damage, and inflammation.^8^^,^^41^ The risk of bias for in vitro studies was assessed with the SYRCLE tool (Figure 4).

**Table 6:** Summary of *in vivo* studies on metformin use in periodontal therapy.

Author	Animal Model	Periodontal Defect Type	MF Form	Delivery Method	Outcomes Measured	Results
Han et al., 2025	Mice	Ligature induced	1:600 (tFNA: MF)	Intraperitoneal	Spectral analysis	Decreases inflammatory cell infiltration, Collagen degradation, and osteoclast Formation, thus Alleviating alveolar bone loss
Xiang et al., 2024	Rats	Ligated stimulated	200 ug	Palatal gingiva injection	Histological analysis and CCK-8 assays	Decreases alveolar bone loss
Zhou et al., 2019	Mice	Streptozotocin induced	200 mg/kg/day	Oral	Micro CT, western blotting, ELISA, and immunofluorescence	Decreases alveolar bone loss and tooth displacement
Araujo et al., 2017	Rats	Ligated stimulated	50, 100, and 200 mg/kg	Oral	Micro CT, immunohistochemical staining, confocal microscopy, UV–vis Analysis, and RT-PCR	Significantly decreases bone loss and inflammation at 50 mg/kg MF
Zhou et al., 2020	Mice	Orally administered p.g. strain	200 mg/kg/day	Oral	Micro CT, ELISA, immunochemistry, and western blotting	Decreases IL-1β and ameliorates inflammation
Ye et al., 2024	Mice	Ligature induced	250 mg/kg/day	Intraperitoneal injection	Western blotting, PCR, and SA-β-gal staining	Alleviates junctional epithelium senescence via the AMPK/SIRT1/autophagy pathway
Sun et al., 2023	Mice	Ligature induced	200 mg/kg	Oral	Micro CT, PCR, ELISA, western blotting, and immunofluorescence	Decreases oxidative stress and tissue destruction by regulating HMGB1 release
Khajuria et al., 2018	Rats	Ligature + p.g. injection	2 mg/kg	Regular inserts	Micro CT, alveolar bone analysis, and histological assessment	Has antibacterial effects and improves alveolar bone properties

Abbreviations: tFNA = Tetrahedral Framework Nucleic Acid, CCK-8 = Cell Counting Kit-8, micro CT = Microcomputed Tomography, ELISA = Enzyme-linked Immunosorbent Assay, UV–vis = Ultraviolet–visible, RT-PCR = Reverse Transcription Polymerase Chain Reaction, p.g = *Porphyromonas gingivalis*, PCR = Polymerase Chain Reaction, SA = β-gal Staining Senescence-associated β-galactosidase.

#### Effects on periodontal ligament stem cells

MF stimulates the release of extracellular vesicles from PDLSCs, thus supporting regeneration of periodontal tissue and osteogenesis.^6^

#### Effects on Immunological and inflammatory pathways

MF has immunomodulatory effects by modulating several key inflammatory pathways, including HMGB1, NLRP3 inflammasomes, and gasdermin D (GSDMD), which are known to contribute to the destruction of periodontal tissue.^9^^,^^42^, ^43^, ^44^

#### Enhancements in drug distribution and bioavailability

To improve the local bioavailability of MF in diabetes animal models, novel drug delivery techniques have been used, with carriers such as trimethyl chitosan and carboxymethylated inferior diameter fiber. These innovative delivery techniques might improve periodontal outcomes, because they have been associated with diminished alveolar bone loss, preservation of the collagen matrix, and increased osteoclastogenesis.^45^

## Discussion

The goal of this review was to evaluate the efficacy of MF as a supplement to surgical and non-surgical periodontal therapy, according to the PICO framework. Across 15 RCTs, MF, compared with conventional or placebo therapy, was found to consistently improve PD, CAL, and bone fill. GRADE assessments showed moderate certainty for PD and CAL improvements and low certainty for radiographic findings, thereby providing reasonable confidence in MF's adjunctive effectiveness.

MF, an insulin-sensitizing medication with cyto-protective properties,^35^ also improves periodontal health through a variety of cellular and molecular processes. RCT findings were compared with in vitro/vivo evidence to identify regions of overlap and divergence, and clarify the biological foundation for clinical results.

Cellular and preclinical research notably supported clinical outcomes in RCTs. In vitro studies indicated that MF inhibits NLRP3 inflammasomes, and decreases IL-1β and IL-18 production,^1^ correlates with diminished inflammation and bleeding on probing.^20^^,^^21^^,^^31^ Gains in CAL and radiographic bone levels in well-contained IBDs^24^^,^^28^ were consistent with findings from animal studies demonstrating decreased osteoclastic activity^35^^,^^39^^,^^41^ and increased osteogenesis via Akt/Nrf2 and AMPK/SIRT1 signaling.^34^^,^^43^ The more robust regeneration observed in animal models was probably due to greater doses or longer treatment,^6^^,^^45^ thus accounting for the diversity in radiographic outcomes among RCTs.

**Figure 5:**
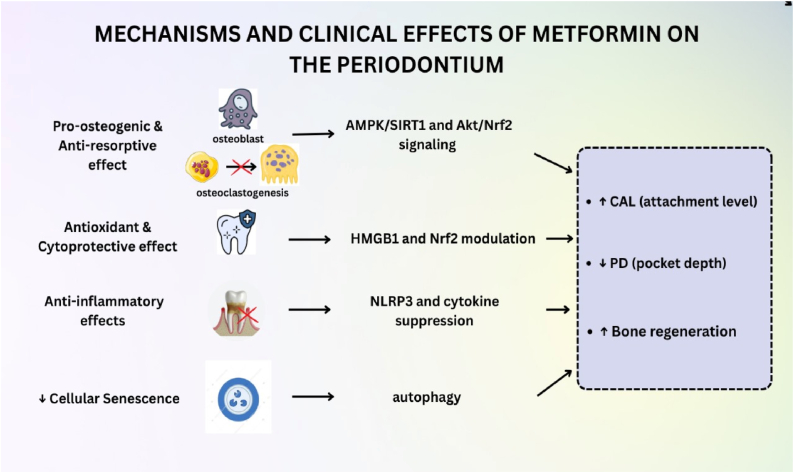
Mechanisms and clinical effects of metformin on the periodontium Metformin promotes osteogenesis and inhibits osteoclastogenesis through AMPK/SIRT1 and Akt/Nrf2 signaling; enhances antioxidant and cytoprotective actions via HMGB1 and Nrf2 modulation; suppresses inflammation by inhibiting NLRP3 and cytokine release; and decreases cellular senescence through autophagy. Collectively, these mechanisms lead to increased clinical attachment level (CAL), reduced probing depth (PD), and enhanced bone regeneration.

Overall, mechanistic and animal studies provided strong support for clinical outcomes and demonstrated that MF's periodontal advantages are due to various biological activities. Its pro-osteogenic and anti-resorptive effects through the AMPK/SIRT1 and Akt/Nrf2 pathways, antioxidant and cytoprotective activity through HMGB1 and Nrf2, anti-inflammatory action through NLRP3 and cytokine suppression, and autophagy-mediated decrease in cellular senescence all contributed to improvements in PD, CAL, and bone regeneration. The mechanisms, including promotion of osteogenesis, inhibition of osteoclastogenesis, antioxidant and cytoprotective effects, anti-inflammatory action, and decreased cellular senescence, and cumulative effects on periodontal healing, are presented in Figure 5.

Clinical evidence indicated that site-specific administration of 1% MF gel, in combination with SRP, improved periodontal outcomes, and achieved greater PD reduction, CAL gain, and radiographic improvement in IBD depth than placebo.^20^, ^21^, ^22^^,^^24^^,^^28^^,^^31^ Although 1.5% MF gel with SRP and curettage has advantages, the 1% formulation produces better outcomes. The gel's mucoadhesive properties enable prolonged retention in periodontal pockets and therefore are beneficial for chronic periodontal disease.^5^

The most consistent results were PD decreases and CAL gains, and radiographic bone fill was also noted. The benefits were stronger in localized IBDs or furcation defects, because of improved medication retention. MF was helpful in both surgical and nonsurgical treatments, but its supplementary effects were more pronounced in surgical patients with well-contained abnormalities. Reports of lower BOP and inflammation were favorable, although variable. The variations in outcomes were probably due to changes in MF formulation, concentration, application period, and baseline illness severity. Overall, MF's effectiveness appeared to be determined more by defect anatomy, plaque reduction, and local medication retention than by dosage alone.

Several systematic reviews and meta-analyses supported these findings, showing positive results for MF as a supplement in periodontal therapy. Local 1% MF has been reported to improve clinical and radiographic parameters in IBDs after 6 months.^46^ When paired with SRP, it decreases the inflammatory burden and potentially the need for additional therapies.^10^^,^^47^ Moreover, it appears to be beneficial in filling bone defects, lowering PD, and increasing CAL.^48^ MF has also been identified as a useful medication for enhancing outcomes in both surgical and non-surgical periodontal therapy.^3^ To our knowledge, this review is among the few to integrate clinical, preclinical, and mechanistic data on MF use in periodontal therapy.

Although the findings revealed consistent clinical and radiographic improvements, several methodological limitations limit their interpretation. Searches excluded databases such as Web of Science, Embase, and Cochrane, and only English-language articles were considered, thus potentially introducing bias. Because significant variability in study design, treatments, disease severity, and follow-up precluded meta-analysis, a narrative synthesis using the SWIM framework was necessary but might have introduced subjectivity. Moreover, the potential for selective outcome reporting cannot be excluded. The variability between surgical and nonsurgical methods hindered accurate estimation of the three key PICO outcomes: CAL gain, PD reduction, and radiographic outcomes, including IBD reduction and MBL.

The existing data had structural limitations, because most RCTs were conducted by several researchers and frequently used numerous sites per patient, thus potentially introducing bias. Future studies should focus on well-powered, multicenter RCTs with defined methods, reliable follow-up, and clear differentiation between localized and widespread illness. Multilevel models that account for systemic variables, smoking, and glycemic management, along with extended follow-up and pre-registered CONSORT-compliant techniques, would increase the reliability and repeatability of the results. Ongoing research has indicated that MF is increasingly used as a periodontal regenerative supplement. Several PROSPERO-registered protocols are assessing 1% MF gel alone or with biomaterials in diverse populations. Examples include evaluation of MF with platelet-rich fibrin (CRD42020216436), with SRP (CRD42017074116), or with regenerative gel application (CRD420250652237); MF's effects on periodontal health (CRD42024542200); and clinical trials (e.g., NCT02274090, NCT03204058, NCT02580331, and NCT06856369). These studies seek to determine optimal concentrations, formulations, and administration routes, as well as MF's long-term effects on bone and attachment regeneration, thereby filling gaps in preclinical data and refining the role of MF in evidence-based periodontal therapy.

## Conclusion

Metformin administered locally, particularly as a gel, enhances the efficacy of traditional periodontal therapy. Clinical experiments demonstrated increased PD, CAL, and bone fill. Preclinical research indicated MF's anti-inflammatory, osteogenic, and cytoprotective properties. However, the small sample sizes, short follow-up periods, and insufficient safety data necessitate larger, standardized, long-term trials to establish efficacy and safety.

## Ethical approval

No ethical approval was required for this systematic review.

## Authors contributions

KH: conceptualization, review, editing, supervision and project administration. AN: conceptualization, methodology, writing original draft, visualization, investigation and data curation. SMA: conceptualization, methodology, writing original draft, visualization, investigation and data curation. SR: conceptualization, methodology, writing original draft, visualization, investigation and data curation. MAS: conceptualization, methodology, writing original draft, visualization, investigation and data curation. MSZ: review and editing, resources, validation. BS: review, editing, resources and visualization. HH: software, review editing, data curation. All authors have critically reviewed and approved the final draft and are responsible for the content and similarity index of the manuscript.

## Source of funding and conflict of interest

This systematic review received no external funding, and has no commercial or financial conflicts of interest to report.
